# Oral mitis group streptococci reduce infectivity of influenza A virus via acidification and H_2_O_2_ production

**DOI:** 10.1371/journal.pone.0276293

**Published:** 2022-11-09

**Authors:** Nobuo Okahashi, Tomoko Sumitomo, Masanobu Nakata, Hirotaka Kuwata, Shigetada Kawabata

**Affiliations:** 1 Center for Frontier Oral Science, Osaka University Graduate School of Dentistry, Suita, Osaka, Japan; 2 Department of Oral and Molecular Microbiology, Osaka University Graduate School of Dentistry, Suita, Osaka, Japan; 3 Department of Oral Microbiology, Kagoshima University Graduate School of Medical and Dental Sciences, Kagoshima, Japan; 4 Department of Oral Microbiology and Immunology, School of Dentistry, Showa University, Shinagawa, Tokyo, Japan; Instituto Butantan, BRAZIL

## Abstract

Members of the mitis group streptococci are the most abundant inhabitants of the oral cavity and dental plaque. Influenza A virus (IAV), the causative agent of influenza, infects the upper respiratory tract, and co-infection with *Streptococcus pneumoniae* is a major cause of morbidity during influenza epidemics. *S*. *pneumoniae* is a member of mitis group streptococci and shares many features with oral mitis group streptococci. In this study, we investigated the effect of viable *Streptococcus oralis*, a representative member of oral mitis group, on the infectivity of H1N1 IAV. The infectivity of IAV was measured by a plaque assay using Madin-Darby canine kidney cells. When IAV was incubated in growing culture of *S*. *oralis*, the IAV titer decreased in a time- and dose-dependent manner and became less than 100-fold, whereas heat-inactivated *S*. *oralis* had no effect. Other oral streptococci such as *Streptococcus mutans* and *Streptococcus salivarius* also reduced the viral infectivity to a lesser extent compared to *S*. *oralis* and *Streptococcus gordonii*, another member of the oral mitis group. *S*. *oralis* produces hydrogen peroxide (H_2_O_2_) at a concentration of 1–2 mM, and its mutant deficient in H_2_O_2_ production showed a weaker effect on the inactivation of IAV, suggesting that H_2_O_2_ contributes to viral inactivation. The contribution of H_2_O_2_ was confirmed by an inhibition assay using catalase, an H_2_O_2_-decomposing enzyme. These oral streptococci produce short chain fatty acids (SCFA) such as acetic acid as a by-product of sugar metabolism, and we also found that the inactivation of IAV was dependent on the mildly acidic pH (around pH 5.0) of these streptococcal cultures. Although inactivation of IAV in buffers of pH 5.0 was limited, incubation in the same buffer containing 2 mM H_2_O_2_ resulted in marked inactivation of IAV, which was similar to the effect of growing *S*. *oralis* culture. Taken together, these results reveal that viable *S*. *oralis* can inactivate IAV via the production of SCFAs and H_2_O_2_. This finding also suggests that the combination of mildly acidic pH and H_2_O_2_ at low concentrations could be an effective method to inactivate IAV.

## Introduction

Many oral streptococci produce short chain fatty acids (SCFAs) such as formic, acetic, and lactic acids as by-products of sugar metabolism [1–4]. Excessive acidification damages the enamel of teeth, and mutans group streptococci such as *Streptococcus mutans* and *Streptococcus sobrinus* are associated with the development of dental caries [1, 2, 4]. *Streptococcus salivarius* is the most abundant streptococcal species in human saliva, and it also produce SCFAs [1–4]. The most abundant inhabitants of dental plaque are mitis group streptococci [1–5]. Oral mitis group streptococci cause a variety of infectious complications such as bacteremia and infective endocarditis [2, 4–6]. This group includes *Streptococcus oralis*, *Streptococcus sanguinis* and *Streptococcus gordonii*. *Streptococcus pneumoniae*, an important pathogen that causes pneumonia, is also a member of the mitis group [6]. These mitis group streptococci produce hydrogen peroxide (H_2_O_2_) in addition to SCFAs [2, 4, 7, 8]. Although the concentration of streptococcal H_2_O_2_ in culture medium is 1–2 mM, it shows an inhibitory effect on the growth of other oral bacteria [7–9], as well as a cytotoxic effect on host innate defense cells [10–12].

Influenza A virus (IAV) infection is a public health problem worldwide [13, 14]. Occasionally, it caused pandemics such as the Spanish flu in 1918, which killed 30–50 million people worldwide. Although IAV alone sometimes causes pneumonia, secondary bacterial infections during and shortly after IAV infection are the most common causes of pneumonia [13–15]. Viral-bacterial pneumonia and secondary bacterial pneumonia strongly influence the morbidity and mortality of IAV infections [13–15].

Poor oral hygiene is reported to be correlated with occurrence of respiratory diseases such as bacterial pneumonia [16], and professional oral healthcare has been shown to reduce the risk of IAV infection [17]. The interaction between IAV and *S*. *pneumoniae* has been intensively investigated [15, 18–21], however, interaction between IAV and oral streptococci is not well understood. Several studies have shown that neuraminidase (NA)-producing streptococci such as *S*. *pneumoniae* and oral mitis group streptococci potentially elevate the risk of influenza because NA plays an essential role in IAV infection [13, 22, 23]. Streptococcal NAs can promote IAV infection and reduce the efficacy of NA inhibitors, such as zanamivir [24] during viral infection.

In addition, mitis group streptococci produce H_2_O_2_. Although the concentration is at the millimolar level, it inhibits the growth of other oral bacteria and hinders the host innate defense system [7, 9–12]. H_2_O_2_ is a strong oxidizing agent, and a 3% solution (equivalent to ~ 1 M) has been used as a disinfectant [25]. It has been reported that 3% H_2_O_2_ effectively inactivate IAV and will be useful for prepare IAV vaccines [26]. It remains unknown whether H_2_O_2_-producing mitis group streptococci influence the infectivity of IAV.

In this study, we found that growing oral mitis group streptococci inactivated IAV *in vitro*. The combination of mildly acidic pH of streptococcal cultures (around pH 5.0) and low concentrations of H_2_O_2_ (around 2 mM) produced by streptococci was able to reduce the infectivity of IAV.

## Materials and methods

### Virus and cell line

IAV A/FM/1/47 (H1N1) [27, 28] were grown in Madin-Darby canine kidney (MDCK) cells as described previously [28, 29]. MDCK cells were cultured in Eagle minimal essential medium [MEM; Invitrogen (Carlsbad, CA, USA)] supplemented with 10% fetal bovine serum (FBS), penicillin (100 U/ml), and streptomycin (100 μg/ml) at 37°C in a 5% CO_2_ atmosphere. Culture supernatants of IAV-infected MDCK cells containing IAV were dispensed and frozen at -80°C. Titers of the IAV frozen stocks were not identical, and varied from experiment to experiment [5 × 10^6^–2 × 10^7^ plaque forming unit (pfu)/ml].

### Bacterial strains and culture conditions

*S*. *oralis* ATCC 35037 [30] was obtained from the Japan Collection of Microorganisms at RIKEN BioResource Center (Tsukuba, Japan). Construction of the *spxB*-deletion mutant (*spxB* KO; deficient in H_2_O_2_ production) from *S*. *oralis* ATCC 35037 wild type (WT) has been described previously [10].

*S*. *gordonii* ATCC 10558, *S*. *salivarius* HHT, *S*. *mutans* MT8148 and *S*. *sobrinus* MT10186 were selected from the stock culture collection of the Department of Oral and Molecular Microbiology, Osaka University Graduate School of Dentistry (Osaka, Japan). *S*. *gordonii* produces H_2_O_2_, whereas *S*. *salivarius*, *S*. *mutans* and *S*. *sobrinus* do not. The bacteria were cultured in brain heart infusion [BHI; Becton Dickinson (Sparks, MD, USA)] broth supplemented with 1% glucose. The BHI broth containing 1% glucose is hereafter referred to as “BHI broth”.

### Incubation of IAV with oral streptococci

Exponential phase cultures of oral streptococci [2 × 10^9^ colony forming units (cfu)] were incubated with IAV (ca. 1–5 × 10^6^ pfu) in 0.5 ml BHI broth at 37°C in a 5% CO_2_ atmosphere. After incubation for 3 h, bacterial growth was stopped by adding penicillin (100 U/ml) and streptomycin (100 μg/ml), and the IAV-bacteria mixture was centrifuged at 3000 × g for 10 min to remove the bacteria. The IAV titer of the supernatants was determined using a plaque assay (see below). The bacterial dose-dependency and time-course change in the IAV titer were measured using *S*. *oralis* WT.

The effects of heat-inactivated *S*. *oralis* WT were also examined. For heat-inactivation, *S*. *oralis* WT was heated at 60°C for 30 min in phosphate-buffered saline (PBS, pH 7.2), centrifuged, and resuspended in BHI broth. Heat-inactivated *S*. *oralis* (equivalent to 2 × 10^9^ to 2 × 10^10^ cfu) was incubated with IAV for 3 h, and the mixture was centrifuged to remove the bacteria. The IAV titer of the supernatants was determined using a plaque assay.

### IAV plaque assay

MDCK cells grown in 6-well culture plates (IWAKI-AGC, Tokyo, Japan) were inoculated with IAV, which was serially diluted 10-fold in 0.1 ml MEM. After adsorption for 1 h, the cells were overlaid with 3 ml of soft agar medium containing MEM (prepared using a powder-type MEM; Nissui, Tokyo, Japan), 0.01% diethylaminoethyl (DEAE)-dextran (Sigma-Aldrich, St. Louis, MO, USA), 2 μg/ml trypsin (Sigma-Aldrich) and 0.8% Agar Noble (Invitrogen), and incubated at 34°C in a 5% CO_2_ atmosphere for 3 days. The infected cells were fixed by 3% formaldehyde in PBS, stained with 0.03% methylene blue solution (Nacalai Tesque, Kyoto, Japan), and the number of plaques was counted [31, 32].

### Effect of pH on IAV infectivity

To estimate the effect of acidification of BHI broth by growing streptococci, BHI broth containing HEPES buffer (0.1 M, pH 7.2; Invitrogen) and phosphate buffer (0.1 M, pH 7.2) were prepared. Exponential phase cultures of streptococci (2 × 10^9^ cfu) were incubated with IAV (ca. 1 × 10^6^ pfu) in these BHI broths (0.5 ml) at 37°C in a 5% CO_2_ atmosphere. After incubation for 3 h, bacterial growth was stopped by adding penicillin and streptomycin, and the mixture was centrifuged to remove the bacteria. The IAV titer of the supernatants was determined using a plaque assay. The final pH of cultures in the stationary phase of growing streptococci in BHI broth was directly measured using pH meter (LAQUA F-71; HORIBA, Kyoto, Japan) after incubation at 37°C in a 5% CO_2_ atmosphere for 18 h.

The effect of acidic pH on IAV inactivation was studied using BHI broth containing sodium acetate (NaOAc) buffer, whose pH was adjusted to 4.0, 4.5, 5.0, or 5.5. IAV in these BHI broth was incubated at 37°C for 3 h, and the viral titer was determined using a plaque assay.

### Effect of H_2_O_2_ on IAV titer

To estimate the contribution of H_2_O_2_ produced by growing *S*. *oralis*, calatase (final 0, 10, 50, and 200 U/ml) was added to BHI broth. Exponential phase cultures of *S*. *oralis* (2 × 10^9^ cfu) were incubated with IAV (ca. 1 × 10^6^ pfu) in 0.5 ml of BHI broth at 37°C in a 5% CO_2_ atmosphere. After incubation for 3 h, bacterial growth was stopped by adding penicillin and streptomycin, and then the mixture was centrifuged to remove the bacteria. The IAV titer of the supernatants was determined using a plaque assay.

To determine the direct effect of H_2_O_2_ on IAV infectivity, IAV in BHI broth was incubated with H_2_O_2_ (0, 1, 2, 5 or 10 mM) at 37°C in a 5% CO_2_ atmosphere. After incubation for 3 h, the IAV titer was determined using a plaque assay. The effect of H_2_O_2_ in BHI broth at pH 5.0 and in MEM (without FBS) on infectivity of IAV was also examined.

### Immunofluorescence

Exponential phase cultures of *S*. *oralis* (2 × 10^9^ cfu) were incubated with IAV (ca. 1 × 10^6^ pfu) in 0.5 ml BHI broth at 37°C in a 5% CO_2_ atmosphere. After incubation for 3 h, bacterial growth was stopped by adding penicillin and streptomycin, and the mixture was centrifuged to remove the bacteria. To investigate direct effect of acidic pH (pH 5.0) and H_2_O_2_ (2 mM) on the infectivity of IAV, BHI broths containing NaOAc buffer (0.1 M; pH 5.0) with or without H_2_O_2_ (2 mM) were prepared. IAV in these BHI broths was incubated at 37°C in a 5% CO_2_ atmosphere for 3 h. MDCK cells grown on Cell Desk LF (Sumitomo Bakelite, Tokyo, Japan) in 24-well culture plates were inoculated with these IAV preparations (50 μl) and incubated at 34°C for 1 h. Then, the culture media containing IAV preparations were discarded, and 1 ml of fresh MEM containing trypsin and DEAE-dextran were added to the wells of the plates. The cells were then incubated at 34°C in a 5% CO_2_ atmosphere for 48 h, fixed with 3% formaldehyde, and permeabilized with 0.5% Triton X-100. The fixed cells were incubated for 30 min with 4’,6-diamidino-2-phenylindole dihydrochloride (DAPI; 0.1 μM) and fluorescein isothiocyanate (FITC)-anti-IAV (1:500 dilution) (Abcam, Cambridge, UK) in PBS containing 1% BSA and 0.1% Triton X 100 at 4°C for 18 h. The fluorescence of the cells was observed using a Carl Zeiss Axioplan 2 fluorescent microscope (Carl Zeiss, Oberkochen, Germany).

### Neuraminidase (NA) assay

IAV NA activity was measured using a neuraminidase assay kit (EnzyChrom neuraminidase assay kit; BioAssay Systems, Hayward, CA, USA). IAV (ca. 1 × 10^6^ pfu) was incubated in phosphate buffer (pH 7.2) and NaOAc buffer (pH 5.0) with or without H_2_O_2_ (2 mM) at 37°C for 3 h. Since a preliminary study showed that H_2_O_2_ and low pH interfered the colorimetric reaction, the IAV suspensions were neutralized with NaOAc (final concentration 0.1 M) and treated with catalase (100 U/ml) at 37°C for 30 min. Each IAV sample (20 μl) was dispensed into 96-well microtiter plates (Sumitomo Bakelite, Tokyo, Japan), mixed with the reaction solution (80 μl) of the assay kit, and incubated at 37°C. Neuraminidase activity was determined as described in the manufacturer’s protocol using a Multiskan FC microplate reader (Thermo Fisher Scientific, Waltham, MA USA).

### Hemagglutinin (HA) assay

IAV (ca. 1 × 10^7^/ml in MEM) was incubated in buffers (phosphate buffer pH 7.2; NaOAc buffer pH 5.0) with or without H_2_O_2_ (2 mM) at 37°C for 3 h. After incubation, the IAV suspensions were neutralized with NaOAc (final concentration 0.1 M) and treated with catalase (100 U/ml) at 37°C for 30 min. Serial two fold dilutions of the viral suspensions (50 μl) were prepared in 96-well round bottom plates (IWAKI-AGC) using PBS. After tadding 50 μl of 5% (v/v in PBS) guinea pig blood (Kojin Bio, Sakado-Saitama, Japan) to each well, the plates were shaken and incubated at 4°C for 1 h. The aggregation of red blood cells was used for determine the titration end point, and the reciprocal of the dilution of the virus was considered to be the HA titer.

### Statistical analysis

Statistical analyses were performed using QuickCalcs software (GraphPad Software, La Jolla, CA, USA) and Ekuseru Toukei (Social Survey Research Information, Tokyo, Japan). Statistical differences were examined using independent Student’s *t*-test. We also compared multiple groups using two-tailed one-way analysis of variance (ANOVA) with Dunnett’s test. A confidence interval with a *p* value of < 0.05 was considered to be significant.

## Results

### Oral streptococci reduce the infectivity of IAV

Infectivity or titer of IAV is usually measured in cell culture media such as MEM. However, our preliminary study showed that growth of oral streptococci is poor in MEM in the absence of FBS. Although FBS enhances the streptococcal growth, it inhibits IAV infectivity. Therefore, in this study, BHI broth was used to investigate the effect of growing streptococci on IAV.

**Fig 1:**
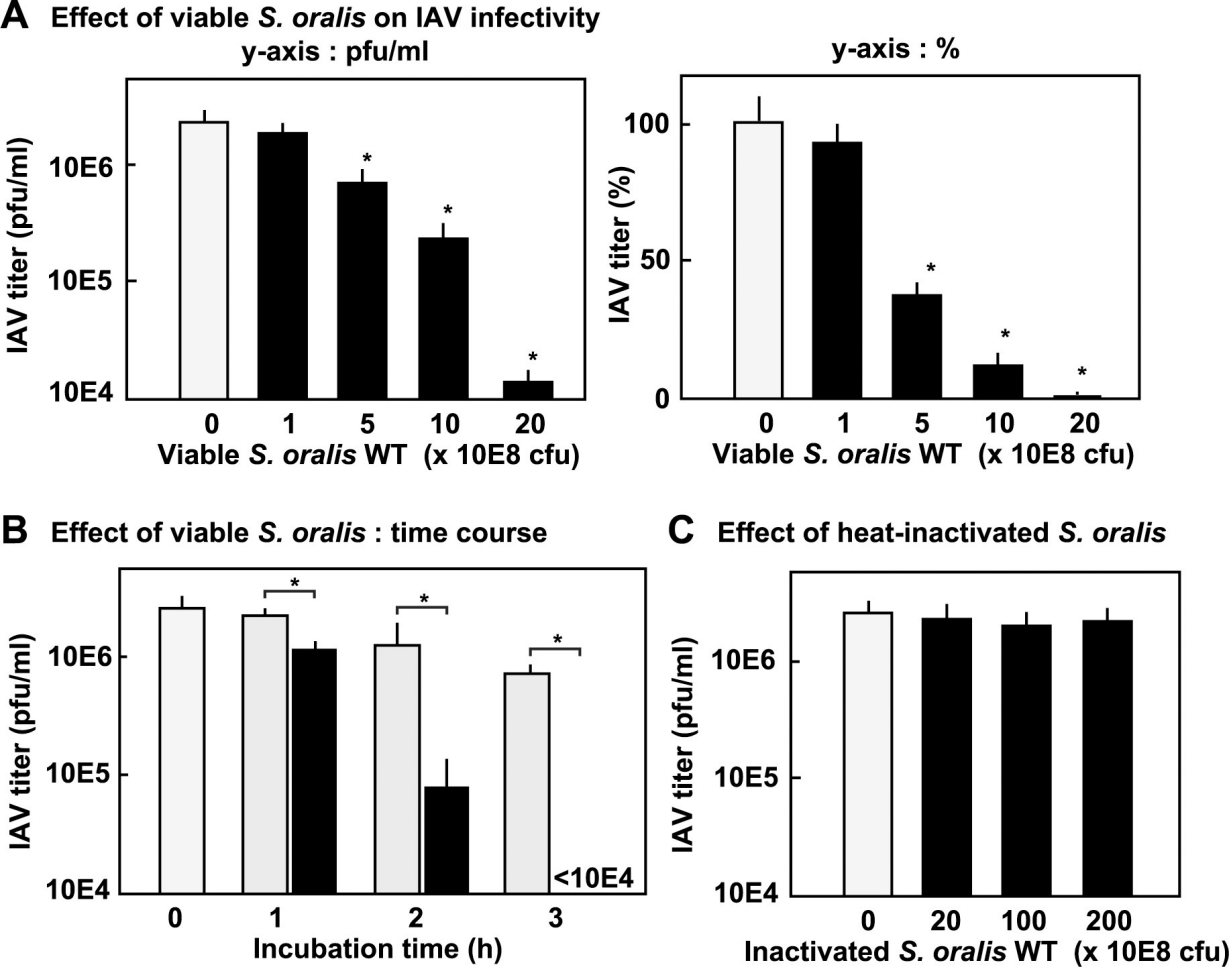
Viable *S*. *oralis* reducs the infectivity of influenza A virus (IAV). (A) IAV in brain heart infusion (BHI) broth was incubated with growing *S*. *oralis* wild type (WT) (1 × 10^8^ to 2 × 10^9^ cfu) at 37°C in a 5% CO_2_ atmosphere. After incubation for 3 h, the bacterial growth was stopped by adding antibiotics, and the IAV-bacteria mixture was centrifuged to precipitate the bacteria. The IAV titer in the supernatants was determined using a plaque assay. The IAV titer was expressed as the pfu/ml (A left) and % of the untreated control IAV (A right). (B) *S*. *oralis* WT was heat-inactivated at 60°C for 30 min in phosphate buffered saline (PBS). The heat-inactivated *S*. *oralis* (equivalent to 2 × 10^9^ to 2 × 10^10^ cfu) was incubated with IAV for 3 h, and centrifuged to precipitate the bacteria. The titer in the supernatants was determined using a plaque assay. (C) IAV in BHI broth was incubated with or without growing *S*. *oralis* WT (2 × 10^9^ cfu) at 37°C for 0, 1, 2, and 3 h. The bacterial growth was stopped by adding antibiotics, and the IAV-bacteria mixture was centrifuged to precipitate the bacteria. The titer was determined using a plaque assay. 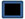: with *S*. *oralis*; 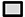: without *S*. *oralis*. The data are shown as mean ± SD values of triplicate samples. *p < 0.05 as compared with the untreated control (no bacteria, or no *S*. *oralis*).

First, we investigated the effect of growing viable *S*. *oralis* WT, which is a representative member of the oral mitis group streptococci, on IAV infectivity. The infectivity of the IAV incubated with growing *S*. *oralis* WT decreased in a dose- and time-dependent manner (Fig 1A and 1B). The percentage representation of the bar graph (Fig 1A right) shows an obvious decrease in the infectivity of IAV. Viable *S*. *oralis* WT (2 × 10^9^ cfu) reduced the infectivity of IAV by 100 times after 3 h (Fig 1A and 1B). Heat-inactivated *S*. *oralis* WT showed no effect on IAV even at a bacterial dose corresponding to 2 × 10^10^ cfu (Fig 1C), indicating that the inactivation of IAV was caused by the growth of viable streptococci.

**Fig 2:**
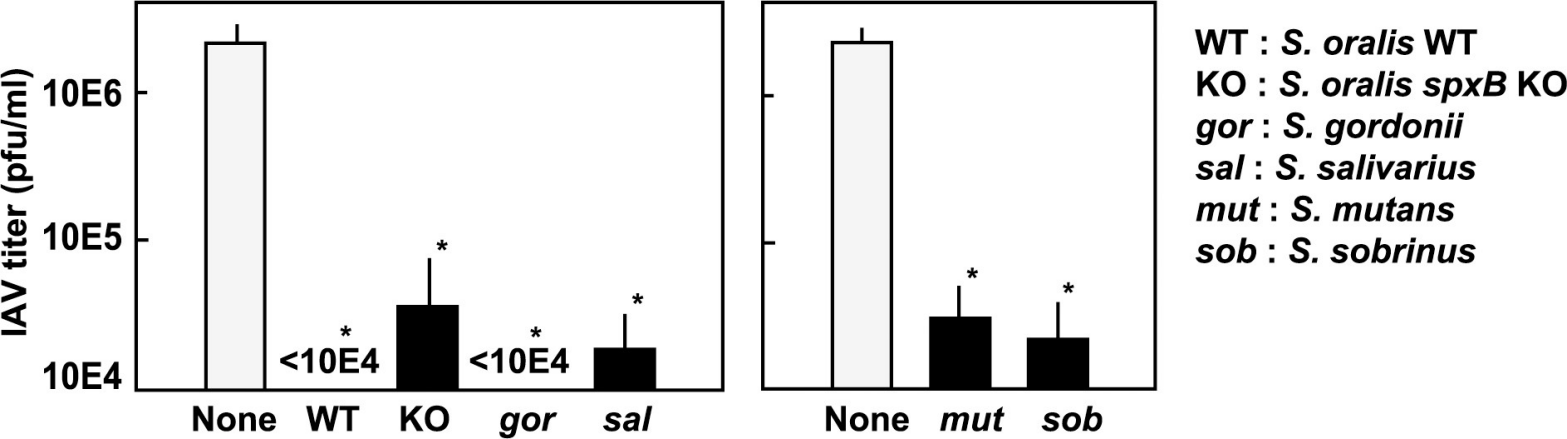
Effect of viable oral streptococci on the infectivity of IAV. IAV in BHI broth was incubated with growing *S*. *oralis* WT (WT), *S*. *oralis spxB* KO (KO), *S*. *gordonii* (*gor*), *S*. *salivarius* (*sal*), *S*. *mutans* (*mut*), or *S*. *sobrinus* (*sor*) (2 × 10^9^ cfu) at 37°C in a 5% CO_2_ atmosphere. After incubation for 3 h, the bacterial growth was stopped by adding antibiotics, and the bacteria were removed by centrifugation. The IAV titer in the supernatants was determined using a plaque assay. The data are shown as mean ± SD values of triplicate samples. *p < 0.05 as compared with the untreated control (no bacteria; None).

**Fig 3:**
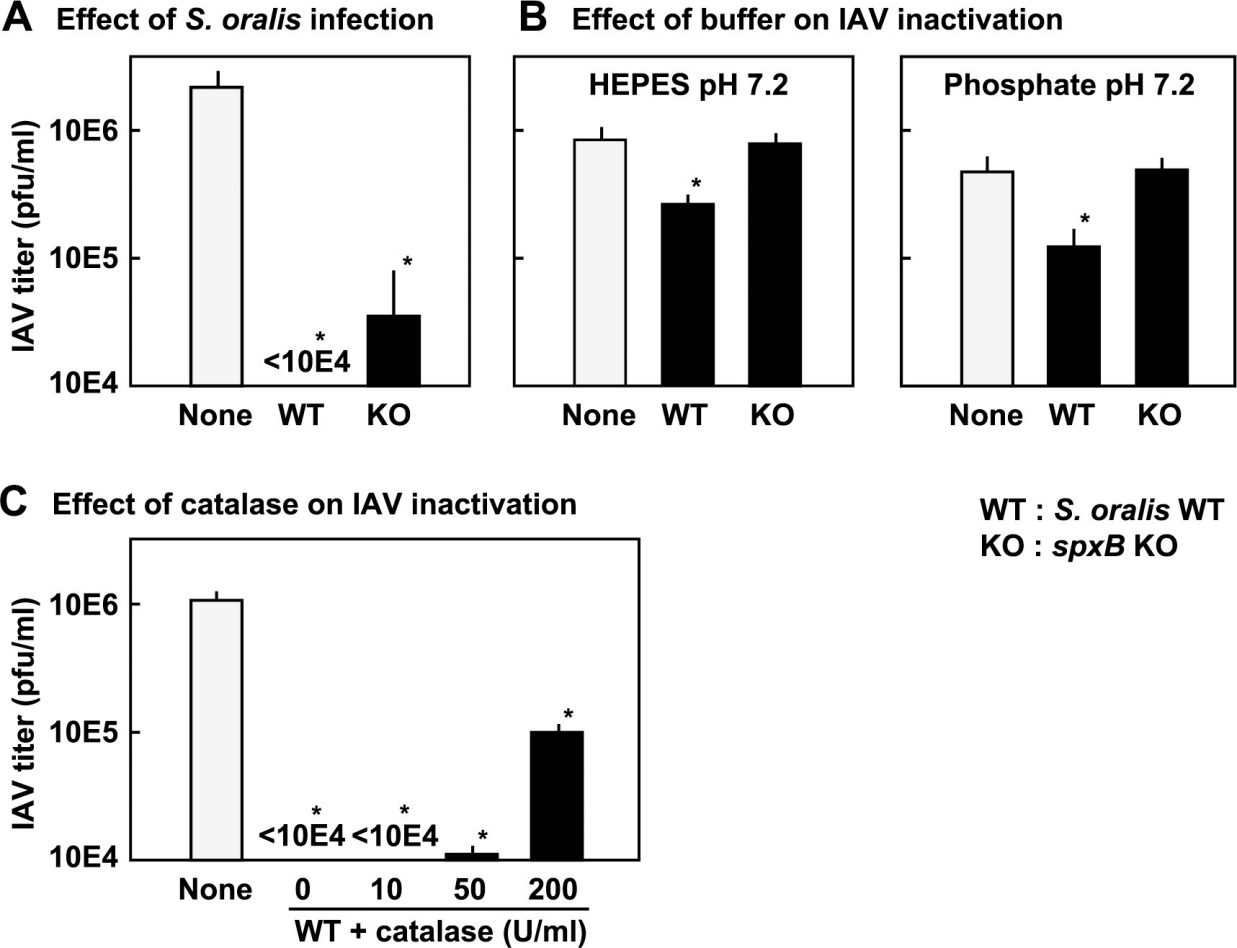
Effect of buffer and catalase on the *S*. *oralis*-induced inactivation of IAV. (A) IAV in BHI broth was incubated with growing *S*. *oralis* WT and *S*. *oralis spxB* KO (2 × 10^9^ cfu) at 37°C in a 5% CO_2_ atmosphere. After incubation for 3 h, the bacterial growth was stopped by adding antibiotics, and the bacteria were removed by centrifugation. The IAV titer in the supernatants was determined using a plaque assay. (B) IAV in BHI broth containing HEPES buffer (0.1 M, pH 7.2; left) or phosphate buffer (0.1 M, pH 7.2; right) was incubated with growing *S*. *oralis* WT and *S*. *oralis spxB* KO (2 × 10^9^ cfu) at 37°C for 3 h. The bacterial growth was stopped by adding antibiotics, and the bacteria were removed by centrifugation. The titer was determined by the plaque assay. (C) IAV in BHI broth containing catalase (0–200 U/ml) was incubated with growing *S*. *oralis* WT (2 × 10^9^ cfu) at 37°C for 3 h. The bacterial growth was stopped by adding antibiotics, and the bacteria were removed by centrifugation. The titer was determined using a plaque assay. The data are shown as mean ± SD values of triplicate samples. *p < 0.05 as compared with the control (no bacteria; None).

Next, the effects of other members of the oral streptococci were investigated. All five species of oral streptococci, *S*. *oralis*, *S*. *gordonii*, *S*. *salivarius*, *S*. *mutans* and *S*. *sobrinus* were shown to reduce the infectivity of IAV (Fig 2). However, the degree of inactivation was not equivalent, and the effects of *S*. *salivarius*, *S*. *mutans* and *S*. *sobrinus* seemed to be weaker than that of H_2_O_2_-producing *S*. *oralis* and *S*. *gordonii*. The bacterial dose-dependency study of *S*. *salivarius* on IAV inactivation showed that the inactivation by *S*. *salivarius* was weaker than that by *S*. *oralis*, suggesting that streptococcal H_2_O_2_ contributed to the viral inactivation (S1 Fig). The inactivating ability of *S*. *oralis spxB* KO mutant, which is deficient in H_2_O_2_ production [10], was also weaker than that of *S*. *oralis* WT (Fig 2 left; see also Fig 3A). Measurement of the H_2_O_2_ concentrations in these streptococcal cultures confirmed that *S*. *salivarius*, *S*. *mutans* and *S*. *sobrinus* did not produce H_2_O_2_ (S2 Fig).

### Effect of buffers and catalase on the inactivation of IAV

Oral streptococci including *S*. *oralis* are known to produce SCFAs such as formic, acetic, and lactic acids as by-products of sugar metabolism, and their cultures become acidic. To assess the contribution of acidification to the inactivation of IAV, HEPES buffer (pH 7.2) or phosphate buffer (pH 7.2) was added to BHI broth, and the infectivity of IAV after incubation with *S*. *oralis* WT or *spxB* KO was measured. In the absence of the buffer, *S*. *oralis* WT reduced the infectivity of IAV by more than 100-fold, while the reduction by *spxB* KO was not complete (Fig 3A; see also Fig 2 left). In the presence of buffers at pH 7.2, inactivation of IAV was not observed in culture of *S*. *oralis spxB* KO, whereas partial inactivation of IAV was still observed in culture of *S*. *oralis* WT (Fig 3B).

These results suggest that streptococcal H_2_O_2_ partially contributed to the reduction in IAV. Therefore, we examined the effect of catalase, an H_2_O_2_-decomposing enzyme, on *S*. *oralis*-induced inactivation of IAV. As shown in Fig 3C, catalase reduced the inactivating effect of viable *S*. *oralis* WT (Fig 3C). Even in the presence of catalase (200 U/ml), the infectivity of IAV was lower than that of the control (Fig 3C). This remaining inactivating effect was considered to be due to acidification by *S*. *oralis* (see Fig 3A and Fig 2 left). Phosphate and HEPES buffers or catalase did not inhibit the streptococcal growth (S3 Fig).

### Effect of pH on the infectivity of IAV

**Fig 4:**
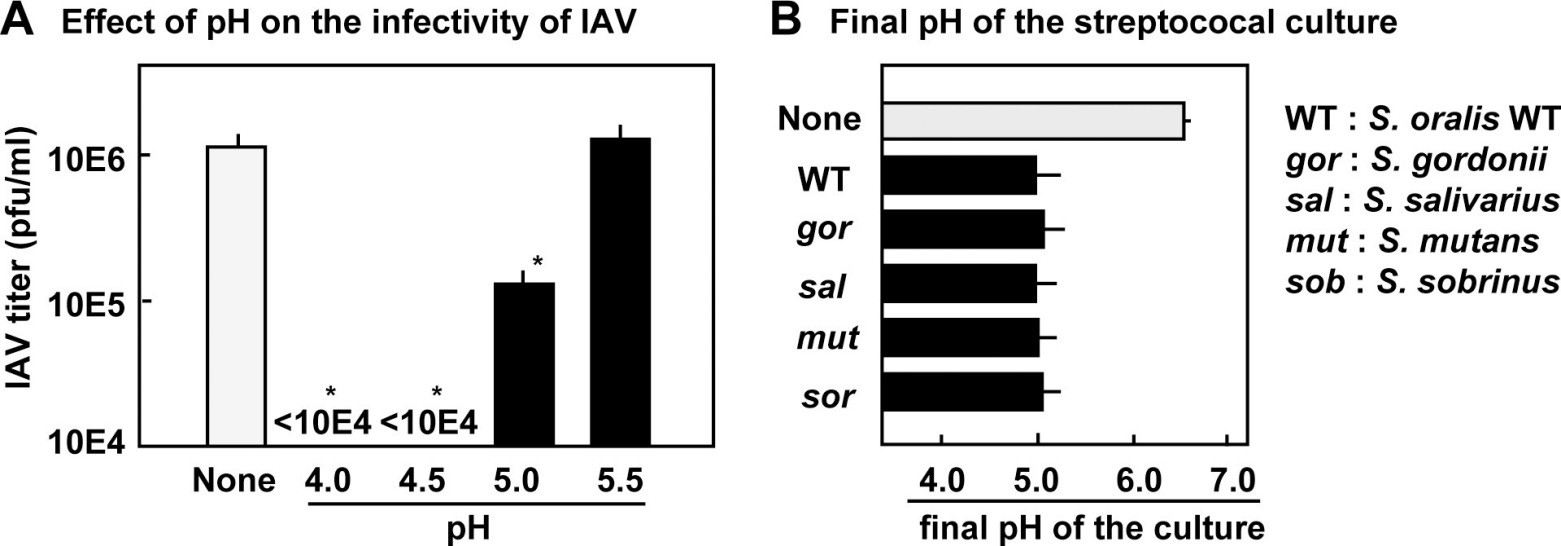
Effect of pH on the infectivity of IAV. (A) Effect of mildly acidic pH on IAV inactivation was studied using BHI broth containing NaOAc buffer (0.1 M; pH 4.0, 4.5, 5.0 and 5.5). IAV in these BHI broth was incubated at 37°C in a 5% CO_2_ atmosphere for 3 h. The titer of the IAV was determined using a plaque assay. The data are shown as mean ± SD values of triplicate samples. *p < 0.05 as compared with the control (None; no NaOAc buffer). (B) Final pH of the streptococcal cultures was measured. *S*. *oralis* WT (WT), *S*. *gordonii* (*gor*), *S*. *salivarius* (*sal*), *S*. *mutans* (*mut*), and *S*. *sobrinus* (*sor*) were cultured in BHI broth at 37°C in a 5% CO_2_ atmosphere for 3 h, as the same condition for the IAV inactivation study. Then, the pH of the cultures was directly measured using a pH meter (LAQUA F-71; HORIBA, Kyoto, Japan).

The direct effect of acidic pH on IAV inactivation was studied using BHI broth containing NaOAc buffer. IAV was incubated in BHI broth at pH 4.0, 4.5, 5.0 and 5.5, and the IAV titer was determined. Fig 4 shows that the infectivity of IAV diminished in BHI broths at pH 4.0 and pH 4.5. No reduction in infectivity was observed in BHI broth at pH 5.5. A partial reduction in infectivity (by approximately 10 times) was observed at pH 5.0. Since these results suggested that acidification played an important role in *S*. *oralis*-induced inactivation of IAV, the final pH of the streptococcal cultures in BHI broth after 3 h was measured (Fig 4B). The final pH of *S*. *oralis* WT was pH 5.1, and that of other streptococcal cultures was between pH 5.2 and pH 5.1.

### Effect of H_2_O_2_ on the infectivity of IAV

**Fig 5:**
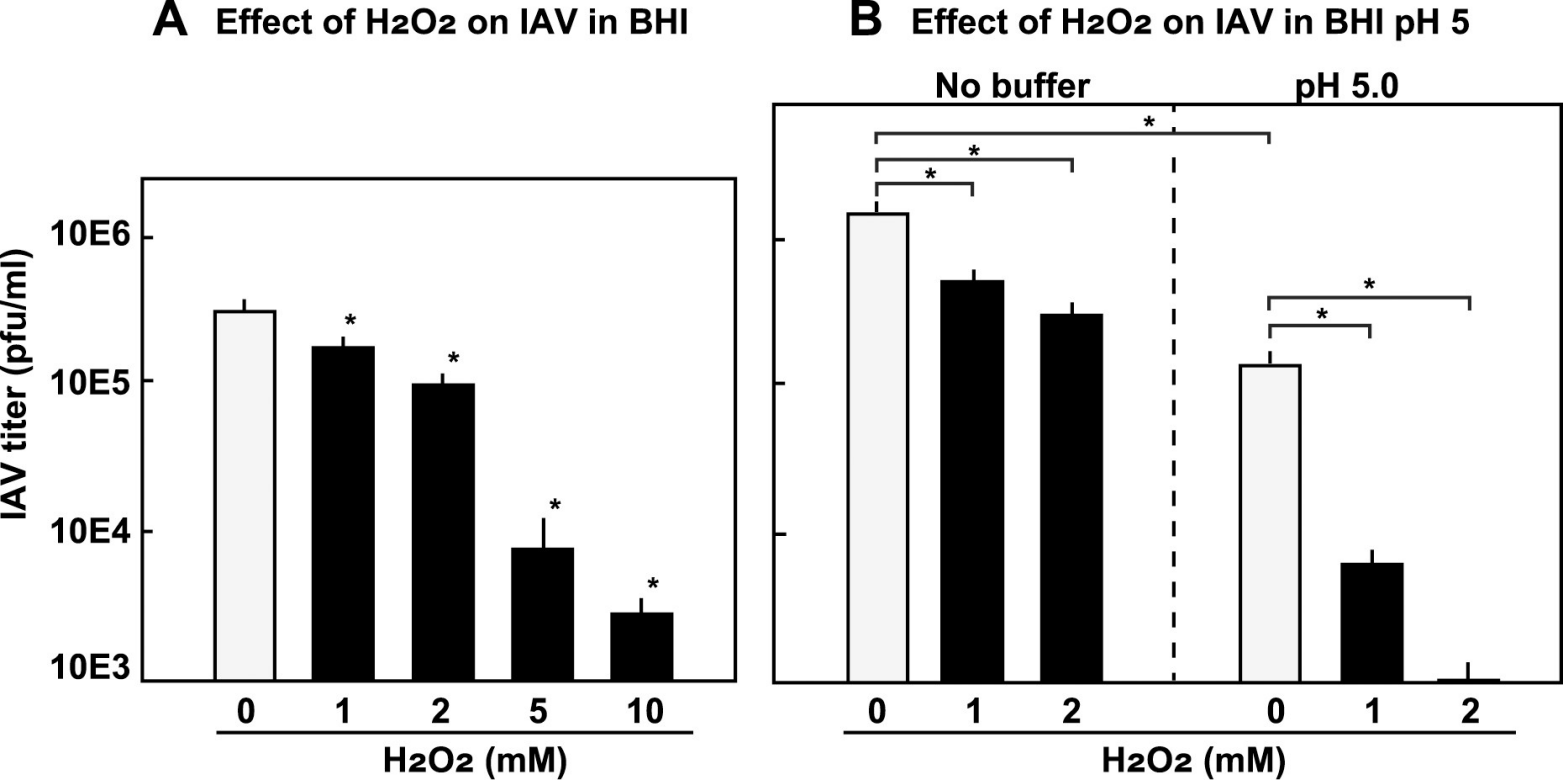
H_2_O_2_ reduces the infectivity of IAV. (A) IAV in BHI broth was incubated with H_2_O_2_ (1, 2, 5, or 10 mM) at 37°C for 3 h. The IAV titer was determined using a plaque assay. (B) IAV in BHI broth with or without 0.1 M NaOAc (pH 5.0) was incubated with H_2_O_2_ (0, 1, and 2 mM) at 37°C for 3 h, and the titer of the IAV was determined using a plaque assay. The data are shown as mean ± SD values of triplicate samples. *p < 0.05 as compared with the untreated control (no H_2_O_2_).

The above results suggest that in addition to the acidification, H_2_O_2_ promoted the inactivation of IAV. Therefore, the direct effect of H_2_O_2_ on IAV infectivity was investigated. In the BHI broth, H_2_O_2_ reduced IAV infectivity in a dose-dependent manner (Fig 5A). In MEM, the inactivating effect of H_2_O_2_ was more obvious, suggesting that BHI broth reduced the effect of H_2_O_2_ (S4 Fig).

Furthermore, the effect of H_2_O_2_ in BHI broth adjusted to pH 5.0 using NaOAc buffer was examined. As shown in Fig 5B, the inactivating effect of H_2_O_2_ on IAV was enhanced in the BHI broth at pH 5.0. These results revealed that both acidification and H_2_O_2_ cooperatively inactivated the IAV.

### Visualization of the infectivity of IAV by fluorescence staining

**Fig 6:**
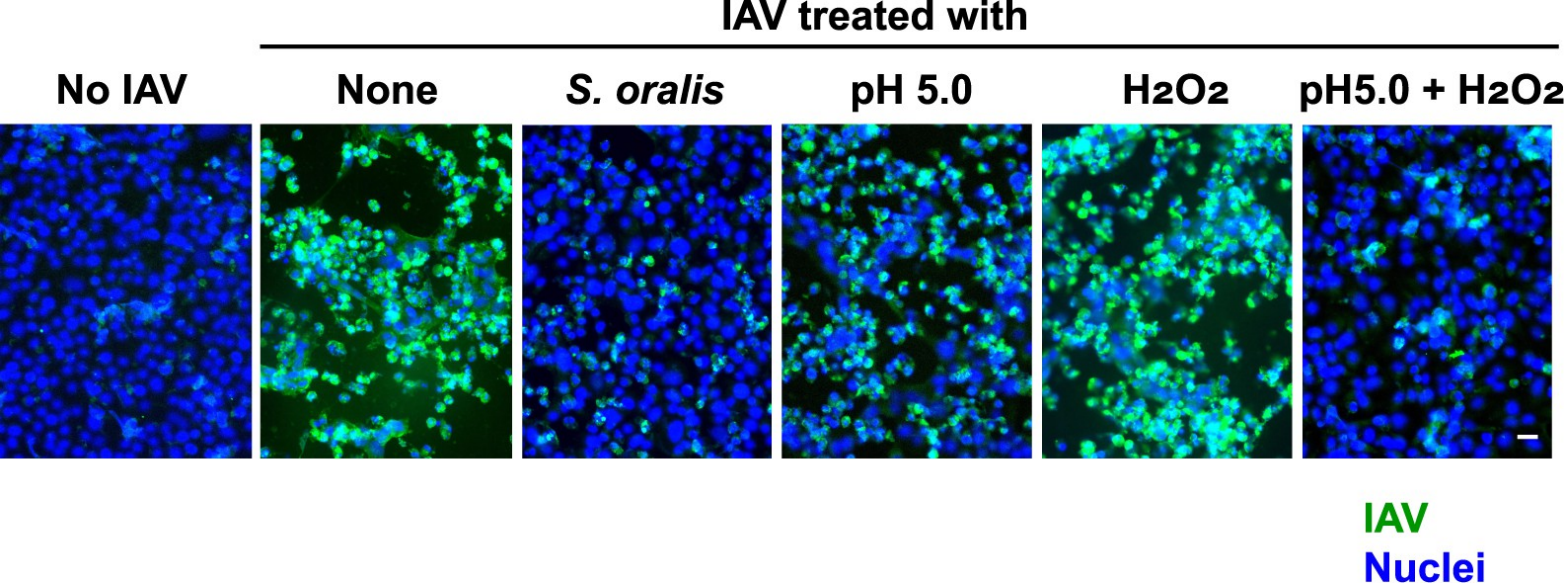
**Fluorescence staining of MDCK cells infected with IAV.** IAV was incubated with viable *S*. *oralis* WT (2 × 10^9^ cfu) in BHI broth at 37°C for 3 h. Other samples of IAV were incubated in broths containing 2 mM H_2_O_2_, or NaOAc buffer (0.1 M; pH 5.0) with or without H_2_O_2_. These IAV preparations (50 μl) were inoculated to the MDCK cells in 24-well culture plates. The cells were fixed, and stained with FITC-anti IAV antibody (Green) and DAPI (Blue). The fluorescence of the cells was observed using a fluorescent microscope system. Bar = 10 μm.

The cell-to-cell spread of IAV was evaluated using immunofluorescence staining. IAV was incubated with viable *S*. *oralis* WT in BHI broth, or in broths containing 2 mM H_2_O_2_, or NaOAc buffer (0.1 M; pH 5.0) with or without H_2_O_2_ for 3 h. MDCK cells were treated with these IAV preparations, and then, the cells were stained with FITC-anti-IAV antibody and DAPI. The IAV-infected cells were visualized as green (Fig 6, None). Some cells were detached because of cell death induced by infection. The reduction in infectivity of IAV incubated with viable *S*. *oralis* WT was confirmed by this staining, and the distribution of green fluorescence was limited (Fig 6, *S*. *oralis*). A partial reduction in green fluorescence was observed in IAV incubated in BHI broth at pH 5.0 (Fig 6, pH 5.0), and a clear reduction was observed in IAV incubated in BHI broth at pH 5.0 containing 2 mM H_2_O_2_ (Fig 6, pH 5.0 + H_2_O_2_). These images confirm that the inactivation of IAV was due to the combined action of acidification and H_2_O_2_.

### Effect of acidic pH and H_2_O_2_ on NA and HA activities of IAV

**Fig 7:**
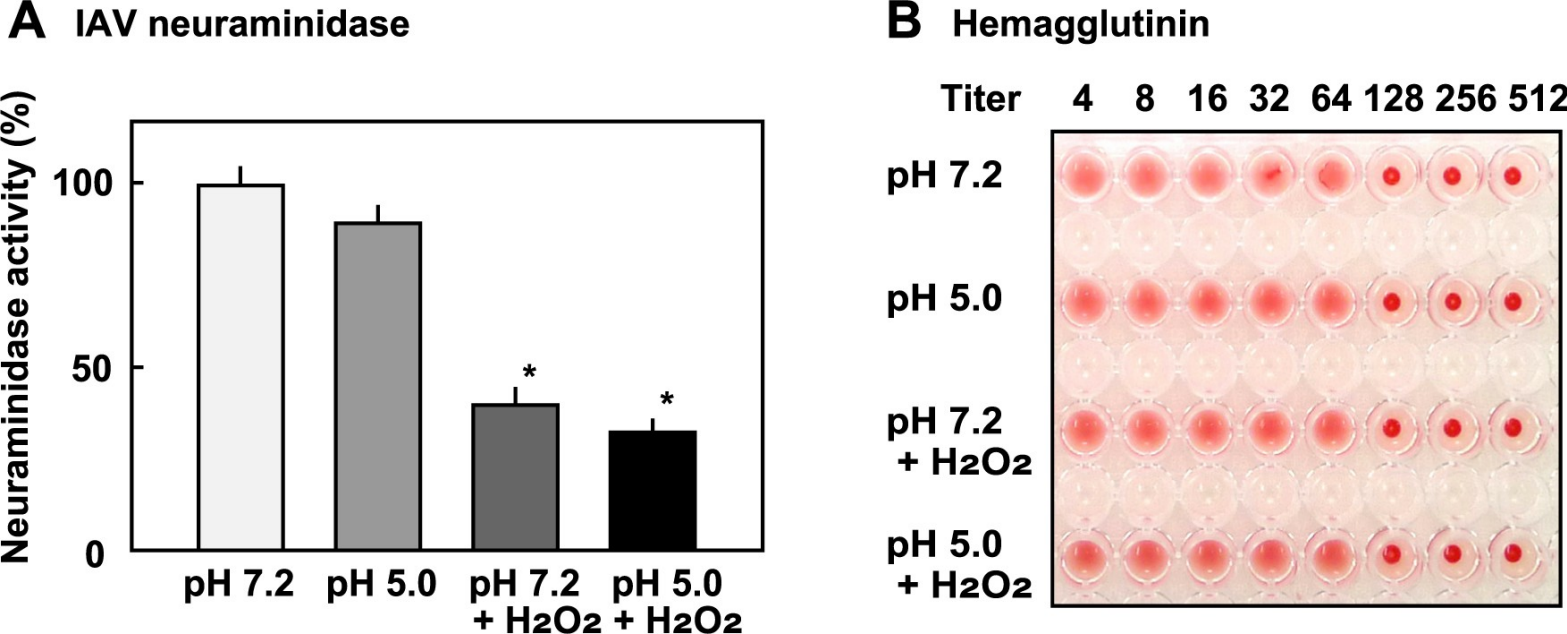
Effect of acidic pH and H_2_O_2_ on neuraminidase and hemagglutinin activities of IAV. (A) IAV in 0.1 M buffers (phosphate buffer, pH 7.2; NaOAc buffer, pH 5.0) with or without H_2_O_2_ (2 mM) was incubated at 37°C for 3 h. Neuraminidase activity of IAV was measured by using a neuraminidase assay kit. (B) IAV was incubated in buffers (phosphate buffer pH 7.2; NaOAc buffer pH 5.0) with or without H_2_O_2_ (2 mM) at 37°C for 3 h. Serial two fold dilutions of the virus suspensions (50 μl) were prepared in 96-well round bottom plates using PBS. After addition of 50 μl of a 5% (v/v in PBS) guinea pig blood to each well, the plates were incubated at 4°C for 1 h, and the visible aggregation of the red blood cells was observed.

It is established that the NA and HA plays an essential role in the infection by IAV [13, 14]. Therefore, the effects of acidic pH and H_2_O_2_ on viral NA and HA was investigated in this study. As *S*. *oralis* produces its own NA, the effect of viable *S*. *oralis* on viral NA could not be investigated. IAV in 0.1 M buffers (phosphate buffer, pH 7.2 and NaOAc buffer, pH 5.0) with or without H_2_O_2_ (2 mM) was incubated at 37°C for 3 h, and viral NA activity and HA activity were measured. NA activity was not influenced by incubation in the buffer (pH 5.0); however, H_2_O_2_ reduced its activity significantly, while the inactivation was not complete (Fig 7A). HA activity was not affected by these treatments (Fig 7B), suggesting that the reduction in the infectivity of IAV at pH 5.0 is not related to the inactivation of HA.

## Discussion

**Fig 8:**
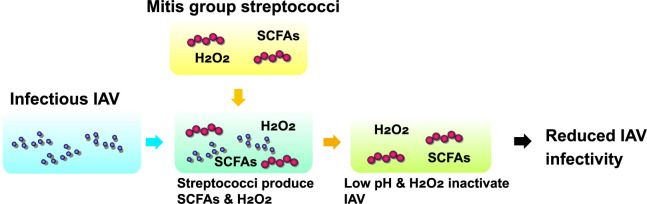
Summary of this study. Mitis group streptococci, such as *S*. *oralis*, produce SCFAs and H_2_O_2_. SCFAs decrease environmental pH, and the combination of mildly acidic pH (around pH 5.0) and H_2_O_2_ (around 2 mM) reduces the infectivity of IAV.

This study revealed that the infectivity of H1N1 IAV was reduced by incubation with growing viable *S*. *oralis*, a member of the oral mitis group streptococci, and the inactivation of IAV was dependent on the combined action of mildly acidic pH (around pH 5.0) and low concentrations of H_2_O_2_ (around 2 mM) in the streptococcal culture. Acidification was due to the production of SCFAs by streptococci. The two streptococcal by-products, SCFAs and H_2_O_2_, cooperatively contributed to the reduction in the NA activity of IAV. The results are summarized in Fig 8.

IAV infects the upper respiratory tract and oral mitis group streptococci are inhabitants of the oral cavity [2, 6, 33–35]. The interaction between IAV and *S*. *pneumoniae*, a pathogenic member of the mitis group, have been intensively investigated [2, 20, 21, 36–39]; however, the interaction between IAV and oral streptococci has not been well studied.

Regarding to the co-infection with IAV, NA produced by the oral mitis group of streptococci are of interest [22, 23, 40]. NA is a glycoside hydrolase that cleave the glycoside linkage of neuraminic acids [13, 14]. Viral NA is found on the surface of IAV and it is an antigenic determinant of IAV [13, 14]. IAV relies on viral NA activity to release progeny viruses from infected cells and spread infection; thus, NA inhibitors such as zanamivir are useful to treat influenza [24]. NA is also produced by oral mitis group streptococci and it is possible that streptococcal NA boosts IAV infection. In fact, two studies [22, 23] have reported the contribution of oral mitis group streptococci to infection by and release of IAV. Culture supernatants of the mitis group streptococci containing NA promoted the release of IAV and cell-to-cell spreading of infection. These studies suggest that NA-producing oral bacteria may increase the risk of the onset and exacerbation of IAV infection. However, our study presented another picture of the interaction between IAV and oral mitis group streptococci. The infectivity of IAV was reduced by viable oral mitis group streptococci, and inactivation of the virus was dependent on acidic pH and H_2_O_2_. Therefore, it cannot be simply considered that co-infection with mitis group streptococci always promotes IAV infection.

Oral mitis group streptococci produce SCFAs as by-products of sugar metabolism [1–4] and broth cultures of these streptococci usually become mildly acidic, reaching pH 4.5–5.0 (see Fig 4B). Low pH reduces the infectivity of IAV. It should be noted that a classical study showed that low pH inactivates IAV. Schiltissek [41] reported that the infectivity of avian H7N1 IAV was rapidly lost (within 30 min) at pH 5.2. His study also showed that the infectivity of H1N1 IAV strains was diminished at pH 5.4–6.0. This pH range was not consistent with our results showing that complete inactivation of IAV occurs at pH below 4.5 (Fig 4A). However, a more recent study by Nishide et al. [42] reported that IAV showed significant inactivation at pH 5.0, with a nearly 10-fold reduction, and complete inactivation was achieved at pH 4.0. Another study showed that IAV was rapidly inactivated by contact with acid-buffered solutions at pH 3.5, and suggested the potential of a low-pH nasal spray as an adjunct to influenza therapies [43]. Acid inactivation has been widely reported in a variety of viruses, such as herpes simplex virus and rhinovirus, and some acidic chemicals have been examined to prevent viral infection [42, 44].

HA is a glycoprotein found on the surface of IAV [13, 14]. It is a fusion protein that is responsible for binding IAV to sialic acid on the surface of target host cells. During viral infection, HA is triggered by endosomal low pH, which causes membrane fusion during viral entry [45, 46]. Recently, the relationship between HA activation and viral inactivation was studied using a luciferase reporter assay [47]. The study concluded that the coupling of HA inactivation and viral inactivation pH was associated with human adaptation [47]. Our study on viral HA suggested that the reduction in infectivity of IAV at pH 5.0 is not related to the inactivation of HA (Fig 7B).

Our study suggests that streptococcal H_2_O_2_ can reduce the infectivity of IAV. In the BHI broth, H_2_O_2_ of low concentrations reduced the infectivity of IAV in a dose-dependent manner, and under acidic conditions, its effect was enhanced (Fig 5). In MEM, the inactivating effect of H_2_O_2_ was stronger (S4 Fig), suggesting that components such as peptides and metal ions in BHI broth reduce the effect of H_2_O_2_. In human oral cavity, saliva has a pH neutralizing effect [1, 3, 4, 38, 48], and the salivary components would reduce the effect of streptococcal H_2_O_2_ [3, 7, 8, 48]. It is not known whether streptococcal by-products, acids and H_2_O_2_, can inactivate IAV in the actual oral cavity as in this study. However, since *Streptococcus* is the most predominant genus in oral cavity, the finding that the metabolic by-products of oral streptococci can inactivate IAV would give new insight on oral ecology. This finding also suggested that the combination of weak acids and hydrogen peroxide of low concentrations will be applicable for prevention of the IAV infection.

H_2_O_2_ is a strong oxidizing agent that has been used as a disinfectant [25]. H_2_O_2_ (3%; equivalent to ~ 1 M) is reported to inactivate many viruses with minimal damage to immunogenicity, and several studies have shown that treatment with H_2_O_2_ can be an effective method for vaccine production [49]. Mice immunized with H_2_O_2_-inactivated West Nile virus were fully protected against lethal challenge [50]. Dembinski et al. [26] demonstrated that IAV is inactivated by 3% H_2_O_2_, and the inactivated IAV retains immunogenicity and can both detect humoral and elicit cellular immune responses *in vitro*.

Taken together, our study revealed that viable H_2_O_2_-producing streptococci, such as *S*. *oralis*, are able to inactivate IAV through production of SCFAs and H_2_O_2_. In addition, our study suggested that H_2_O_2_ of low concentrations in mildly acidic solutions can be useful for preventing IAV infection. Chemicals, such 70% ethanol, are commonly used to inactivate IAV [42, 51, 52]. However, we think that H_2_O_2_ of low concentrations in mildly acidic solutions would lead to alternative methods to reduce the infectivity of IAV.

## Supporting information

S1 Fig IAV-inactivation by H_2_O_2_-non producing *S. salivarius*. Exponential phase cultures of *S*. *salivarius* (0–2 × 10^9^ cfu) were incubated with IAV (ca. 2 × 10^6^ pfu) in 0.5 ml BHI broth for 3 h at 37°C in a 5% CO_2_ atmosphere. After incubation, bacterial growth was stopped by adding antibiotics, and the IAV-bacteria mixture was centrifuged to remove the bacteria. The IAV titer of the supernatants was determined using a plaque assay.
(PDF)

S2 Fig H_2_O_2_ production by oral streptococci. *S*. *oralis* WT (WT), *S*. *gordonii* (*gor*), *S*. *salivarius* (*sal*), *S*. *mutans* (*mut*), or *S*. *sobrinus* (*sor*) were cultured in BHI broth as the same condition for IAV-inactivation study. After incubation for 3 h, the H_2_O_2_ concentrations of the culture supernatants were determined using a hydrogen peroxide colorimetric assay kit (ENZO Life Sciences, NY, USA). The data are shown as mean ± SD values of triplicate samples.
(PDF)

S3 Fig Streptococcal growth in BHI broth containing phosphate buffer, HEPES buffer, or catalase. BHI broth containing HEPES buffer (0.1 M, pH 7.2), phosphate buffer (0.1 M, pH 7.2), or catalase (200 U/ml) was prepared (see Fig 3B & 3C). *S*. *oralis* WT were incubated in these BHI broths as the same condition for the IAV-inactivation study. After incubation for 3 h, the cultures were diluted by PBS, and the absorbance at OD_550_ was determined using a spectrophotometer. The data are shown as mean ± SD values of triplicate samples.
(PDF)

S4 Fig Effect of H_2_O_2_ on infectivity of IAV in MEM medium. (A) IAV in BHI broth was incubated with H_2_O_2_ (1, 2, 5, or 10 mM) at 37°C for 3 h. The IAV titer was determined using a plaque assay. (B) IAV in MEM was incubated with H_2_O_2_ at 37°C for 3 h, and the titer was also determined. The IAV titer was expressed as a % of the untreated control IAV, and the data are shown as mean ± SD values of triplicate samples. *p < 0.05, compared to the untreated control (no H_2_O_2_).
(PDF)

S1 Appendix Minimal data set. Values used to build graphs were listed in these sheets.
(PDF)

S2 Appendix Original images used in Fig 6. (PDF)

S3 Appendix List of the abbreviations. (PDF)
