# Supplementary material for: Oral mitis group streptococci reduce infectivity of influenza A virus via acidification and H2O2 production
Source: PLoS One. 2022 Nov 9;17(11):e0276293. doi: 10.1371/journal.pone.0276293 (PMC9645635; doi:10.1371/journal.pone.0276293)
Supplement: S1 Fig — Exponential phase cultures of S. salivarius (0–2 × 10^9^ cfu) were incubated with IAV (ca. 2 × 10^6^ pfu) in 0.5 ml BHI broth for 3 h at 37°C in a 5% CO2 atmosphere. After incubation, bacterial growth was stopped by adding antibiotics, and the IAV-bacteria mixture was centrifuged to remove the bacteria. The IAV titer of the supernatants was determined using a plaque assay. (PDF) [file pone.0276293.s001.pdf]

S1 Fig Okahashi et al

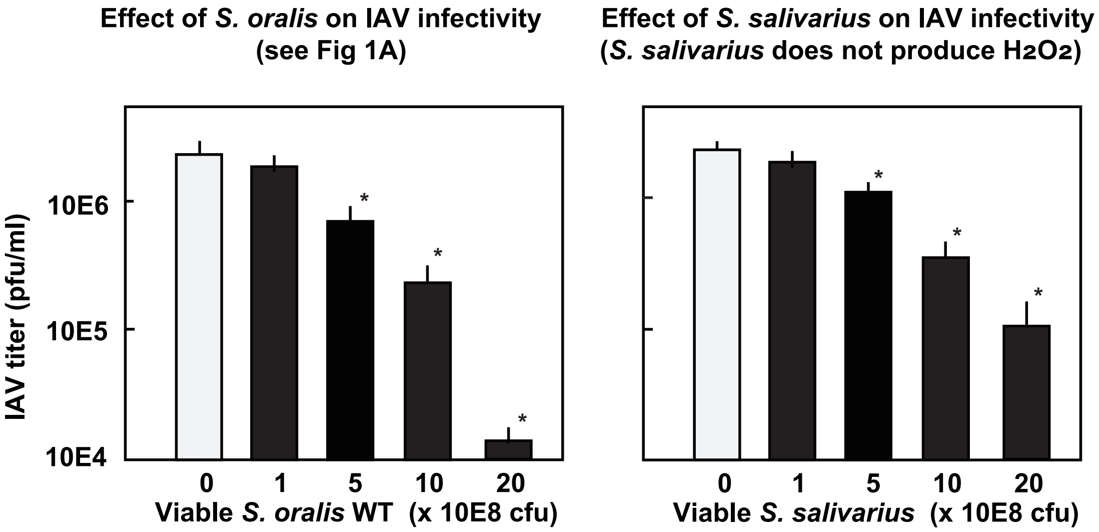

Exponential phase cultures of *S. salivarius* (0 - 2 × 10^9 cfu) were incubated with IAV at 37 °C in a 5% CO_2 atmosphere. After incubation for 3 h, bacterial growth was stopped by adding antibiotics, and the IAV-bacteria mixture was centrifuged to remove the bacteria. The IAV titer of the supernatants was determined using a plaque assay.

The results showed that although *S. salivarius* could inactivate IAV, its effect was weaker than that of *S. oralis*. The difference may be due to the production of H_2O_2.
