# Supplementary material for: Oral mitis group streptococci reduce infectivity of influenza A virus via acidification and H2O2 production
Source: PLoS One. 2022 Nov 9;17(11):e0276293. doi: 10.1371/journal.pone.0276293 (PMC9645635; doi:10.1371/journal.pone.0276293)
Supplement: S2 Fig — S. oralis WT (WT), S. gordonii (gor), S. salivarius (sal), S. mutans (mut), or S. sobrinus (sor) were cultured in BHI broth as the same condition for IAV-inactivation study. After incubation for 3 h, the H2O2 concentrations of the culture supernatants were determined using a hydrogen peroxide colorimetric assay kit (ENZO Life Sciences, NY, USA). The data are shown as mean ± SD values of triplicate samples. (PDF) [file pone.0276293.s002.pdf]

S2 Fig Okahashi et al.

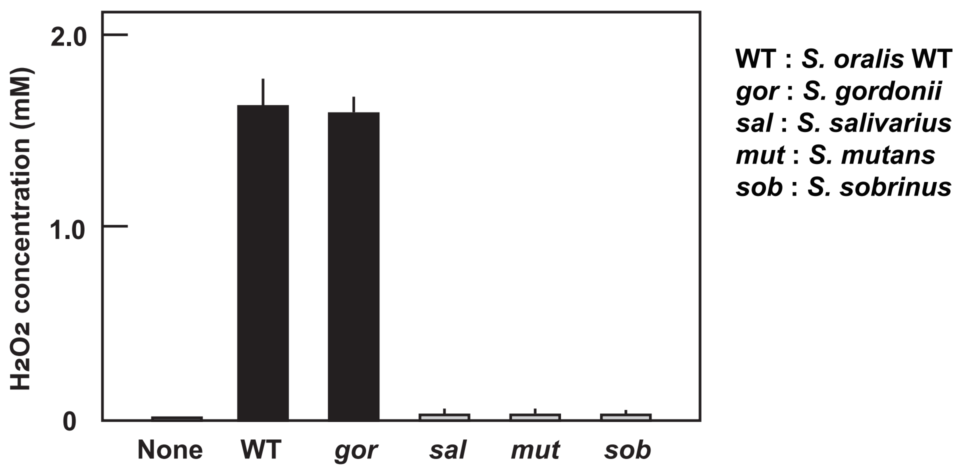

S. oralis WT (WT)*, S. gordonii* (*gor*)*, S. salivarius* (*sal*)*, S. mutans* (*mut*), or *S. sobrinus* (*sor*) (2 × 10^9 cfu) were cultured in BHI broth as the same condition for IAV-inactivation study (see Fig 2). After incubation for 3 h, the H_2O_2 concentrations were measured.

The results suggested that the H_2O_2 concentraitons of *S. oralis* and *S. gordonii* cultures reached to around 1.7 mM under this culture condition. *S. salivarius*, *S. mutans* and *S. sobrinus* did not produce H_2O_2.
