# Supplementary material for: Oral mitis group streptococci reduce infectivity of influenza A virus via acidification and H2O2 production
Source: PLoS One. 2022 Nov 9;17(11):e0276293. doi: 10.1371/journal.pone.0276293 (PMC9645635; doi:10.1371/journal.pone.0276293)
Supplement: S3 Fig — BHI broth containing HEPES buffer (0.1 M, pH 7.2), phosphate buffer (0.1 M, pH 7.2), or catalase (200 U/ml) was prepared (see Fig 3B & 3C). S. oralis WT were incubated in these BHI broths as the same condition for the IAV-inactivation study. After incubation for 3 h, the cultures were diluted by PBS, and the absorbance at OD550 was determined using a spectrophotometer. The data are shown as mean ± SD values of triplicate samples. (PDF) [file pone.0276293.s003.pdf]

S3 Fig Okahashi et al.

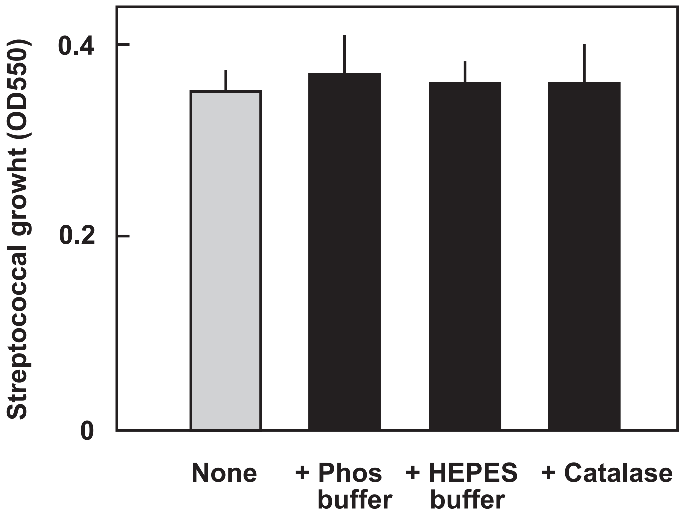

S.oralis WT was grown in BHI broth containing phosphate buffer (pH 7.2), HEPES buffer (pH 7.2), or catalase (200 U/ml) for 3 h as the same condition for the IAV-inactivation study. The cultures were diluted to 20-fold in PBS, and the OD550 was measured.

No significant difference in streptococcal growth was detected. The densities of these cultures were equivalent to about 1.5 x 10^{10} cfu/ml.
