# Supplementary material for: Oral mitis group streptococci reduce infectivity of influenza A virus via acidification and H2O2 production
Source: PLoS One. 2022 Nov 9;17(11):e0276293. doi: 10.1371/journal.pone.0276293 (PMC9645635; doi:10.1371/journal.pone.0276293)
Supplement: S4 Fig — (A) IAV in BHI broth was incubated with H2O2 (1, 2, 5, or 10 mM) at 37°C for 3 h. The IAV titer was determined using a plaque assay. (B) IAV in MEM was incubated with H2O2 at 37°C for 3 h, and the titer was also determined. The IAV titer was expressed as a % of the untreated control IAV, and the data are shown as mean ± SD values of triplicate samples. *p < 0.05, compared to the untreated control (no H2O2). (PDF) [file pone.0276293.s004.pdf]

S4 Fig Okahashi et al.

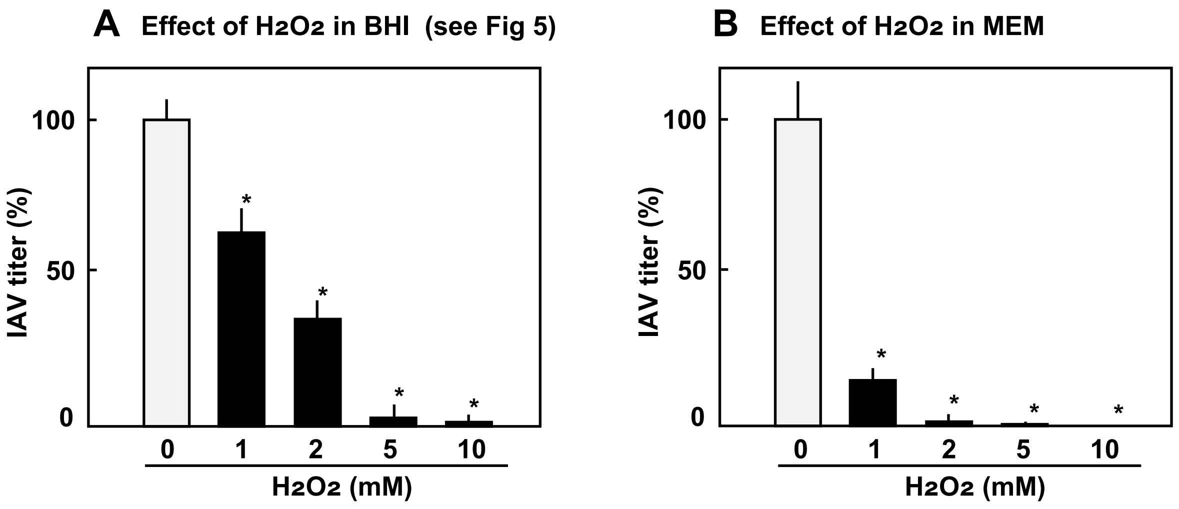

(A) IAV in BHI broth was incubated with H_2O_2 at 37°C for 3 h. The IAV titer was determined using a plaque assay. (B) IAV in MEM was incubated with H_2O_2 at 37 °C for 3 h, and the titer was also determined.

The results showed that inactivating effect of H_2O_2 on IAV in BHI broth is weaker than that in MEM. BHI broth seemed to inhibit the effect of H_2O_2.
