# Supplementary material for: Oral mitis group streptococci reduce infectivity of influenza A virus via acidification and H2O2 production
Source: PLoS One. 2022 Nov 9;17(11):e0276293. doi: 10.1371/journal.pone.0276293 (PMC9645635; doi:10.1371/journal.pone.0276293)
Supplement: S1 Appendix — Values used to build graphs were listed in these sheets. (PDF) [file pone.0276293.s005.pdf]

S1 Appendix Minimal data set. Values used to build graphs

Original values for Figures

Fig 1A

Sample		Dilution	No. of plaque/0.1 ml		
None		1x10E-4	25	20	22
S. oralis	1x10E8	1x10E-4	16	19	22
	5x10E8	1x10E-3	53	69	71
	1x10E9	1x10E-3	29	23	36
	2x10E9	1x10E-2	11	23	16

Fig 1B

Sample		Dilution	No. of plaque/0.1 ml		
None		1x10E-4	24	25	22
heated S. oralis	2x10E9	1x10E-4	18	24	23
	1x10E10	1x10E-4	22	19	20
	2x10E10	1x10E-4	20	23	21

Fig 1C

Sample		Dilution	No. of plaque/0.1 ml		
0 h	None	1x10E-4	23	30	28
1 h	None	1x10E-4	19	21	18
	S. oralis	1x10E-4	15	12	15
2 h	None	1x10E-4	9	12	14
	S.oralis	1x10E-3	5	7	11
3 h	None	1x10E-4	11	8	8
	S. oralis	1x10E-3	0	1	0

Fig 2 left

Sample	Dilution	No. of plaque/0.1 ml		
None	1x10E-4	24	20	29
S. oralis WT	1x10E-3	0	0	0
spxB KO	1x10E-3	4	3	6
S. gordonii	1x10E-3	0	0	0
S. salivarius	1x10E-3	1	1	3

Fig 2 right

Sample	Dilution	No. of plaque/0.1 ml		
None	1x10E-4	24	25	22

S. mutans	1x10E-3	6	1	1
S. sobrinus	1x10E-3	2	3	0

Fig 3A

Sample	Dilution	No. of plaque/0.1 ml		
None	1x10E-4	24	20	29
S. oralis WT	1x10E-3	0	0	0
spxB KO	1x10E-3	4	3	6

Fig 3B

Sample	Dilution	No. of plaque/0.1 ml		
HEPES				
None	1x10E-3	63	72	71
S. oralis WT	1x10E-3	25	26	20
spxB KO	1x10E-3	65	70	72
Phosphate				
None	1x10E-3	36	41	39
S. oralis WT	1x10E-3	15	18	13
spxB KO	1x10E-3	40	44	51

FIg 3C

Sample	Dilution	No. of plaque/0.1 ml		
None	1x10E-3	121	103	112
S. oralis + no catalase	1x10E-1	0	0	3
S. oralis + catalase 10 U/ml	1x10E-1	4	2	2
S. oralis + catalase 50 U/ml	1x10E-2	11	5	8
S. oralis + catalase 200 U/ml	1x10E-2	96	106	108

Fig 4A

Sample	Dilution	No. of plaque/0.1 ml		
None	1x10E-4	11	12	10
pH 4.0	1x10E-3	0	0	0
pH 4.5	1x10E-3	0	0	1
pH 5.0	1x10E-3	14	13	16
pH 5.5	1x10E-4	13	12	12

Fig. 4B

Bacterial culture	Final pH		
None	6.71	6.65	6.66
S. oralis WT	5.06	5.01	5.15

S. gordonii	5.22	5.18	5.11
S. salivarius	5	5.18	5.09
S. mutans	5.04	5.09	5.16
S. sobrinus	5.2	5.13	5.1

Fig 5A

Sample		Dilution	No. of plaque/0.1 ml		
None		1x10E-3	28	31	34
H2O2	1 mM	1x10E-3	16	20	22
	2 mM	1x10E-3	11	11	6
	5 mM	1x10E-3	2	0	0
	10 mM	1x10E-2	2	4	0

Fig 5B

Sample		Dilution	No. of plaque/0.1 ml		
No buffer					
None		1x10E-4	18	12	15
H2O2	1 mM	1x10E-3	47	65	58
	2 mM	1x10E-3	27	34	38
pH 5.0					
None		1x10E-3	9	14	18
	1 mM	1x10E-2	8	5	10
	2 mM	1x10E-2	0	3	1

Fig 7A

Sample	OD550		
Blank	0.063	0.062	0.065
pH 7.2	0.192	0.184	0.177
pH 5.0	0.205	0.184	0.177
pH 7.2 + H2O2	0.128	0.123	0.112
pH 5.0 + H2O2	0.122	0.107	0.108
