# Supplementary figures and images for: Oral mitis group streptococci reduce infectivity of influenza A virus via acidification and H2O2 production
Source: PLoS One. 2022 Nov 9;17(11):e0276293. doi: 10.1371/journal.pone.0276293 (PMC9645635; doi:10.1371/journal.pone.0276293)

## S2 Appendix Original figures for Fig 6

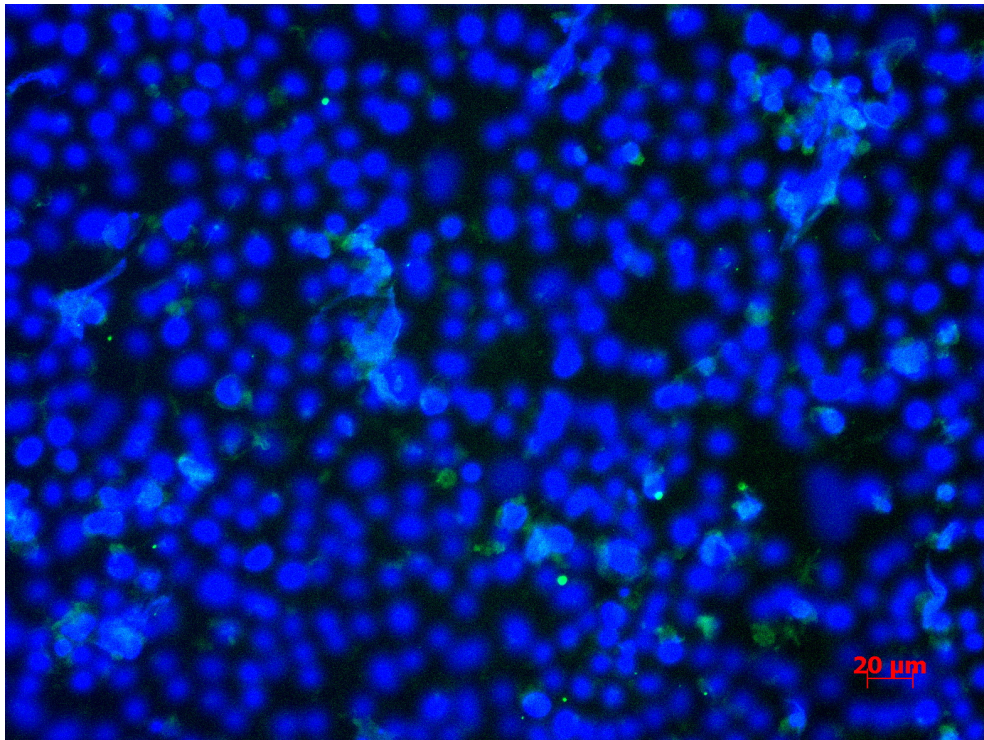

No IAV

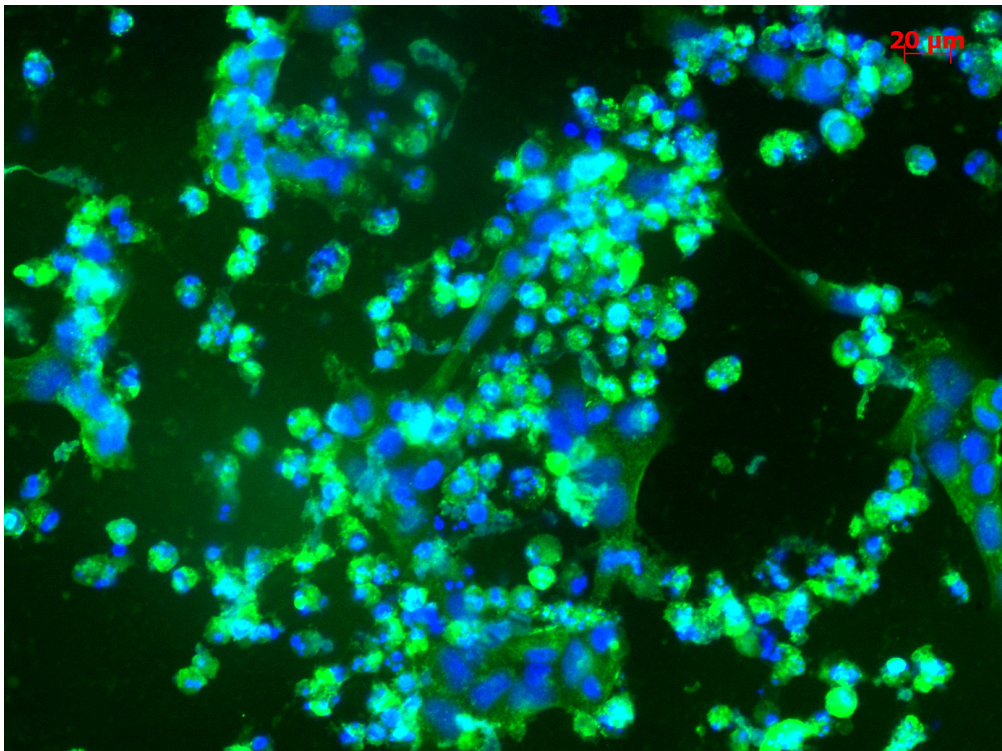

None

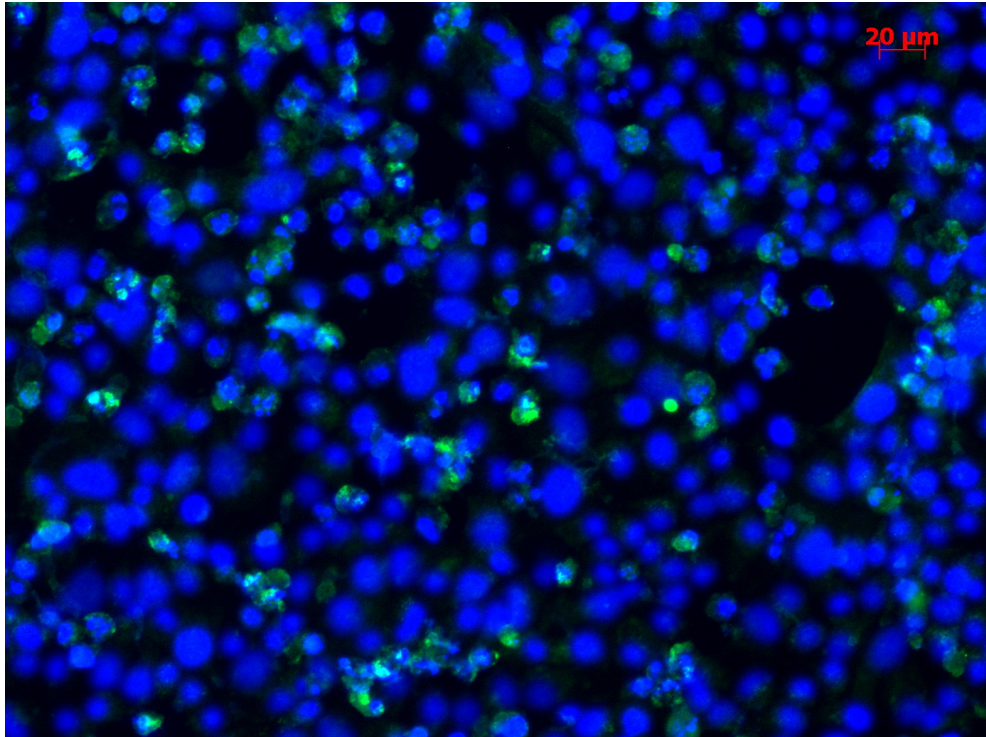

*S. oralis*

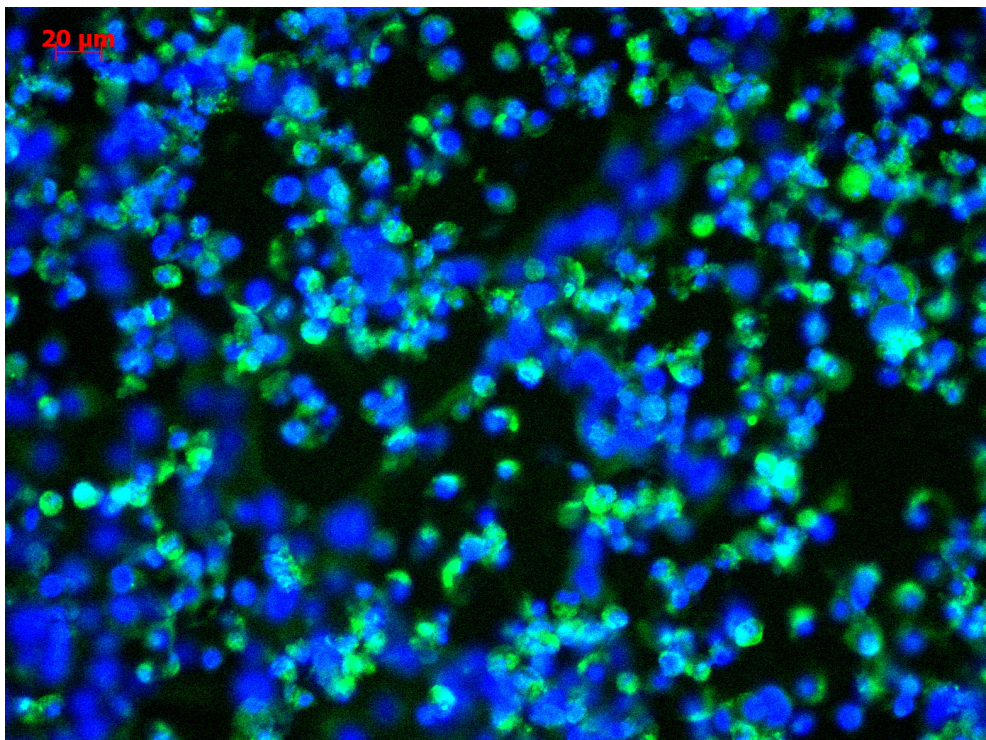

pH 5.0

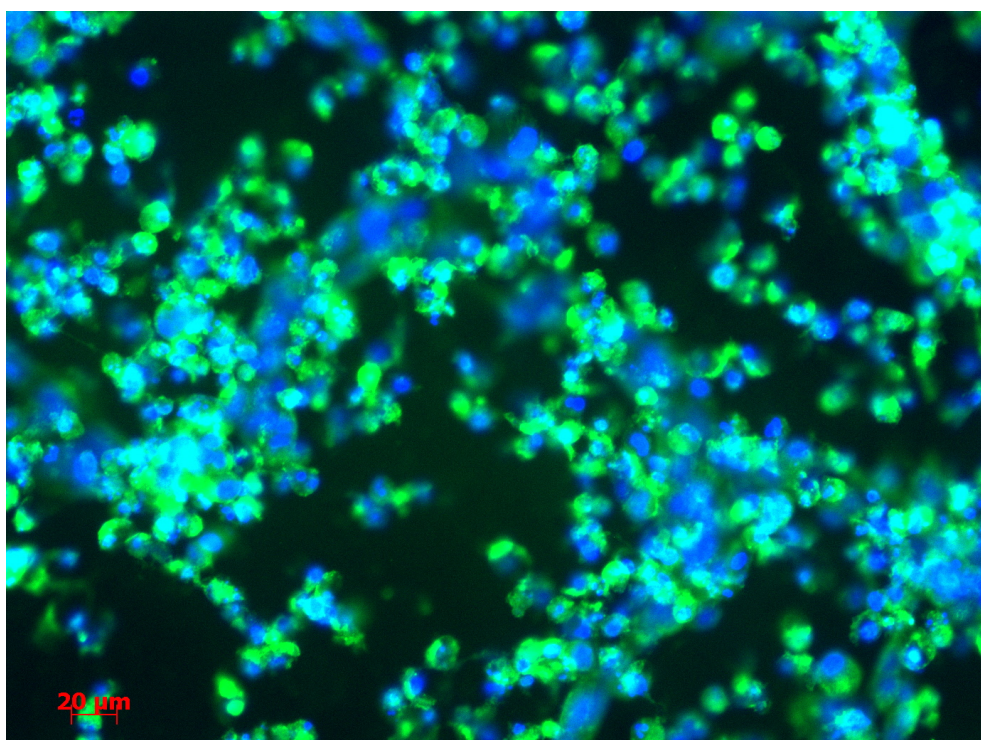

H<sub>2</sub>O<sub>2</sub>

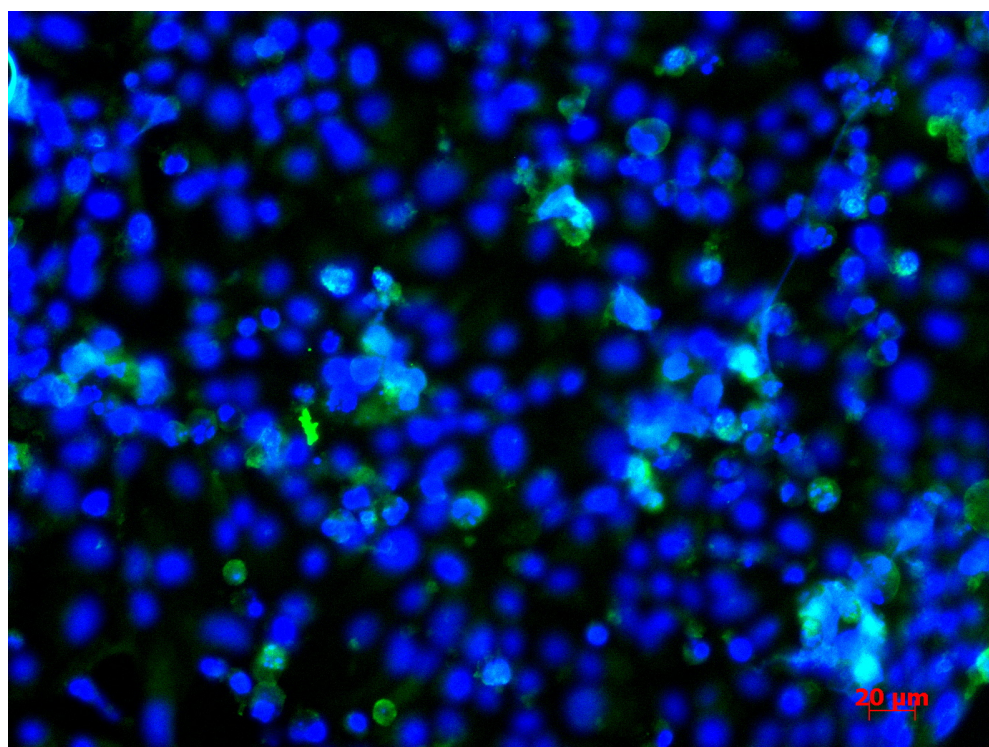

pH 5.0 + H<sub>2</sub>O<sub>2</sub>

Supplement: S2 Appendix — (PDF) [file pone.0276293.s006.pdf]
