# Supplementary material for: Oral mitis group streptococci reduce infectivity of influenza A virus via acidification and H2O2 production
Source: PLoS One. 2022 Nov 9;17(11):e0276293. doi: 10.1371/journal.pone.0276293 (PMC9645635; doi:10.1371/journal.pone.0276293)
Supplement: S3 Appendix — (PDF) [file pone.0276293.s007.pdf]

S3 appendix List of abbreviations

In the text

SCFA, short chain fatty acid; H_2O_2, hydrogen peroxide; IAV, influenza A virus; NA, neuraminidase; MDCK, Madin-Darby canine kidney; MEM, Eagle minimal essential medium; FBS, fetal bovine serum; pfu, plaque forming unit; WT, wild type; BHI, brain heart infusion; cfu, colony forming unit; PBS, phosphate buffered saline; DEAE, diethylaminoethyl; HEPES, 2-[4-(2-hydroxyethyl)-1-piperazinyl]ethanesulfonic acid; NaOAc, sodium acetate, DAPI, 4',6-diamidino-2-phenylindole dihydrochloride; FITC, fluorescein isothiocyanate; HA, hemagglutinin;

In the figures

WT, *S. oralis* wild type strain; KO, *S. oralis spxB*-deficient strain; *gor, S. gordonii, sal, S. salivarius, mut, S. mutans; sor, S. sobrinus*
